# Evaluation of a Telemedicine System for the Transmission of Morpho/Immunological Data Aiming at the Inclusion of Patients in a Therapeutic Trial

**DOI:** 10.1155/2009/767145

**Published:** 2009-01-26

**Authors:** Jean-François Lesesve, Richard Garand

**Affiliations:** Centre Hospitalier Universitaire de Nancy et de Nantes (CHU Nancy et Nantes), 54000 Nancy, France

## Abstract

Due to their high levels of achievement and efficiency, image digitalization and teletransmission tools are more and more frequently used. Applied to cellular haematology, these tools often contribute to diagnosis confrontation, sometimes within the framework of therapeutic trials. We present one of the first approaches of the use of telehaematology for the inclusion of patients in the GOELAMS chronic lymphocytic leukaemia 98 trial. The advantages were (1) the creation of a unique, protected, stable data bank that could be remotely consulted, (2) the use of digitized pictures which made expertise on identical documents possible, (3) the facility of computer exchanges between experts, in terms of reception as well as replying time delays. We were able to set out new standards of image sampling for CLL, solve the semantic divergences, and point out interobserver variability as regards morphology. The limiting factors were the important need for expert investment, but they more importantly concerned the first line morphologists who should benefit from adequate tools, in terms of computer equipment as well as members of staff, so as to apprehend this second reading system as a quality control procedure.

## 1. Introduction

“Expert” reviewing of microscopic data is a well-known procedure in medical practice, within the framework of cooperative studies with therapeutic, epidemiological, or scientific purposes. In the current state of things, this notion, however, mostly remains a theoretical one owing to persisting practical difficulties in its implementation [1]. Telehaematology consists in sending pictures of camera-digitized cells from one computer to another via the internet network. It appears as something easy which is more and more resorted to. Teletransmission of microscopic images enables us to overcome the usual obstacles usually met with traditional methods of smear reviewing (transporting delays, glass slide breakages) and offers new theoretical advantages (above all standardization of the observed cells) [2]. In practice, this teletransmission system remains underused in multicentric studies. Three GOELAMS protocols (Groupe Ouest-Est d’Etude des Leucémies Aiguës et autres Maladies du Sang—Western/Eastern group for the study of acute leukaemias and other blood pathologies) have been completed: our study on chronic lymphocytic leukaemia (CLL) and two other ones on acute myeloid leukaemias. The GOELAMS CLL 98 protocol is being achieved; our goal was to develop the advantages and drawbacks of telehaematology for patient inclusion.

## 2. Materials and Methods

### 2.1. Patients

The GOELAMS CLL 98 study is a randomized multicentric study that compared the effectiveness and tolerance of an intensive treatment with autologous bone marrow transplantation versus CHOP Binet treatment as first-line treatment in patients under 60 years of age, with stage B or C CLL [3]. 86 patients were included on the following criteria: blood lymphocytosis > 15 × 10^9^/L or > 5 × 10^9^/L for at least 3 months, cytologic and histologic medullary infiltrate ≥ 30%, stage B and C, between 18 and 60 years of age, no preliminary treatment or chlorambucil only for less than 6 months.

### 2.2. Methods

May Gründwald Giemsa stained blood smears were sent to an haematological expert located either in Nancy (JFL) or in Nantes (RG) according to the geographic location of the center where the patients had been recruited, France having been for that purpose arbitrarily divided into two parts (East and West). The first expert captured the digital images of the cells and then sent those pictures to the second expert via a teletransmission device. The experts were supposed to have at their disposal the complete blood count and the immunophenotypes performed by the recruiting center and sent to them either by post or via the internet network (after having been scanned) in an attachment. The aim was to obtain a fast second reading of the results and provide the first expert with feedback, the consensus eventually being transmitted to the recruiting center. The morphologic documents were saved in a digitized visual-data bank, the smears could thus quickly be sent back to the labs they initially came from.

The digital images were captured using an optical photonic microscope at ×1000 magnification, an analogic tri CCD camera, a computer connected to the Internet network and to a secure web site (where the digitized pictures could be collected by the first expert and where the second one could receive the files) (TRIBVN and CRIHAN systems). The pictures of the lymphoid cells frequently corresponded to an almost continuous sampling, supposedly representative of the blood smears (ghost cells and poor quality pictures having been removed). Each and every cell was described, different percentages could thus be obtained (mature cells, cleaved cells, lymphoplasmocytoid variants, etc.) and, following from this, morphologic classification of the CLL (common or atypical).

The recruiting center's opinion as well as that of the first and second experts as regards cytology and the interpretation of the immunophenotypes by flow cytometry were codified thanks to a thesaurus which enabled standardization of the vocabulary used by the different specialists as well as improved interpretation of the results (additional thesaurus for haematology of the ADICAP code; Association pour le Développement de l’Informatique en Cytologie et en Anatomopathologie—Association for the Development of Computer science in Cytology and Anatomopathology), regarding the morphology: typical CLL H400; atypical CLL H 401 (mixed prolymphocytic), H402 (mixed pleomorphic), H403 (other cytology, plasmocytoid).

## 3. Results

### 3.1. Workable Files

The duration of the protocol was approximately 7 years (1st review: 29/November/1999; last review: 05/January/2006). 86 patients were included but we could only work on 79-patient data. Some files were indeed not provided by the laboratories, either because they had failed to send us the requested documents (5 patients) or because data had been lost owing to laboratory relocation (2 patients). 17 files were incomplete—with some parameters missing such as the date of validation, the lymphocytosis, the cytologic/immunological data. 56 files were digitized in Nancy and 23 in Nantes.

### 3.2. Agreements between Experts

#### 3.2.1. Morphology (figure 1)

Overall agreement was obtained for 72% of the files (42 cases of typical CLL and 4 atypical). In one case, the three morphologists agreed to exclude the patient (non-Hodgkin lymphoma). Disagreements were reported in 28% of the cases. The diagnosis itself was not challenged, only the exact morphologic classification being at stake (CLL subtypes, “minor” disagreements). Disagreements between the opinions of the recruiter and both experts mainly consisted of reclassification of atypical towards typical CLL (five cases out of eight) or differences regarding the morphologic subtype of atypical CLL (two cases). In one case, the experts changed the CLL subclass from atypical to typical. Disagreements between the experts themselves occurred in 14% of the cases, the first one having classified 6 CLL out of 9 as typical whereas the second had identified them as atypical. In 3 out of 9 CLLs, the two experts did not agree on the morphologic subtype of atypical CLL. In another case, utter disagreement (three diverging opinions) concerned a file that had initially been identified as typical CLL and then changed to atypical, the two experts disagreeing on its morphologic subtype.

As a conclusion, all three agreed on a majority of files and disagreed on minor aspects of a minority of cases. Morphologic agreement is thus possible to achieve. Cytologic classification can consequently definitely be regarded as a reliable and repeatable tool. Last but not least, as for “difficult” cytologies, the method opened the door to discussion, which had not really been the case up to then (the specialist being isolated in their centre and the expert difficult to reach timely).

#### 3.2.2. Immunophenotype (figure 2)

The results of the immunophenotypes were sent to the first expert by the recruiting centre along with the blood smears. Everything (the immunophenotypes and the digitized pictures) was then transferred to the second expert, either by e-mail in an attachment or by post, with a one-day time-lag. The histograms were re-interpreted, and Matutes scores were calculated. All patients had a Matutes score of 4 or 5, except for 2 cases with a score of 3 and one case with a score of 1 (not considered as CLL). Overall agreement between the 3 observers was 86%, including the 3 patients with a score ≤3. Disagreements dawned in 14% of the files. In 4% of the cases, the recruiter's opinion was different from the experts' point of view whereas in 10% of cases, the experts disagreed, one of them however concurring to the recruiter's viewpoint. Disagreements were related to the calculation of the score, between 4 (2 observers) and 5 (1 observer) or vice-versa. The CLL diagnosis was thus never questioned, whatever the morphology. There was no utter disagreement (three diverging opinions). We could thus conclude that as far as immunophenotypical diagnosis is involved, global consensus is not out of reach.

### 3.3. Methodology

We aimed to assess its feasibility (in terms of efficiency, practical side, etc.).

#### 3.3.1. Number of Digitized Cells per Digitized Pictures and per Files (figures 3–5)

**Figure 3:**
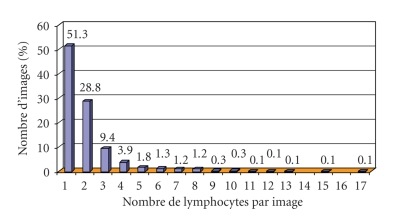
Distribution of the number of lymphocytes per image.

**Figure 4:**
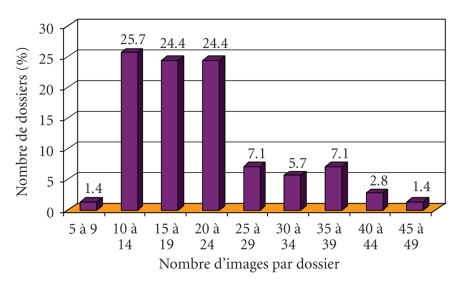
Distribution of the number of images per file.

**Figure 5:**
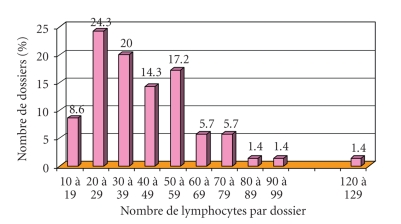
Distribution of the number of lymphocytes per file.

This criterium is essential for the feasibility of this method (review is quicker when cell concentration is higher) and quite a number of cells have to be analyzed before giving one's opinion. The total number of images acquired in this protocol was 1460, consisting of 2938 lymphocytes, which (theoretically) represents an average of 2 cells per picture. The minimum number of lymphocytes captured per image was 1 and the maximum 17, which corresponded to a 508 × 10^9^/L lymphocytosis, namely the highest concentration that could be found in our series (Figure 3). The minimum number of pictures per file was 9 and the maximum 48 (Figure 4). The minimum number of captured lymphocytes for one file was 15 and the maximum 121 (corresponding to the 508 G/L lymphocytosis, Figure 5), the median being 38. 42 lymphocytes per file was the theoretical average (2938 photographed lymphocytes/70 files). Consequently, photographing around 40 lymphocytes per patient seemed appropriate to us as regards this type of lymphoproliferative syndrome. This figure enables the observer taking the pictures to provide the others with a sampling representative of the blood smear and the expert can thus reach a relevant diagnosis.

#### 3.3.2. Number of Lymphocytes Photographed and Complexity of the Morphologic Diagnosis (figure 6)

The CLLs were classified either as typical (code ADICAP H400) or atypical morphology (H401, H402, H403). We chose to focus only on the cases where both experts had a similar cytologic diagnosis (*N* = 53). The average number of lymphocytes captured per file was 40 (±21, *N* = 40) for typical CLL versus 46 (±13, *N* = 13) for atypical CLL. The difference in averages between the typical and atypical CLLs was not significant (*P* > .2, student's *t*-test). The number of digitized lymphocytes is thus not significantly higher when the morphology is atypical.

#### 3.3.3. Cytologic Agreement/Disagreement and Number of Lymphocytes Photographed (figure 7)

The average number of lymphocytes captured per file was 41 (±19, *N* = 53) when both experts were in agreement and 42 (±10, *N* = 10) when they disagreed. The difference between the averages of captured lymphocytes was not relevant (*P* > 0.2, student's *t*-test). The number of captured cells is consequently of no influence on cytologic agreement.

#### 3.3.4. Lymphocytosis and Number of Photographed Images (figure 8)

A link between the lymphocytosis and the number of digitized images could be established (linear Pearson coefficient, −0.46). The higher the lymphocytosis, the less images were taken. This is an important aspect as regards the practical side, since less time was necessary to save and maybe review the documents.

#### 3.3.5. Delays of File Reception (figure 9)

In most cases, the cytologic and immunological documents were sent to one of the experts by post, who then looked after their digitization. Two cases were transmitted directly by e-mail (contrary to what was mentioned in the protocol). In 30% of the cases, the files were received within one month. For the remaining 70%, the time delay was several months and sometimes even reached years! The delay between the two experts using the teletransmission device was less than two weeks in 70% of the cases and 84% of the files were validated within one month of receipt. However, 16% of the files were validated after more than one month (16 weeks being the maximum). Telehaematology enables validation to be completed approximately 12 times faster than the traditional way of review (by post).

## 4. Discussion

This study aimed at putting forward one of the first approaches of the use of telehaematology for the quality control of diagnosis [4]. The GOELAMS CLL 98 trial was chosen to assess this second reading system (blood smears and immunophenotypes). Above all, we wanted to question the efficiency of the method in use since second reading through resort to digitized pictures is, contrary to what might be assumed, a technique which remains largely underused [5].

As regards the morphologic classification, we noticed unanimous agreement in more than 2/3 of the cases. When the morphology was typical, agreement on the cytologic conclusion was almost systematic. 8 cases of disagreement have been reported—the experts having an opinion diverging from the recruiting centre. This can be due to the limited experience of the latter in the field, which justifies the request for a second expert opinion for the inclusion of patients. Furthermore, the experts themselves disagreed on 9 cases. This might seem weird in so far as each digitized cell was analyzed by both experts to classify the CLL. It proves that the same cell can be classified differently by two different specialists. Cytology is above all a matter of interpretation, thus implying a potentially important bias. On the whole, the three specialists agreed on most cases; and the experts disagreed on minor aspects of a minority of cases (the CLL diagnosis was never challenged). Immunophenotypical profile of the CLLs is the second important means to classify the illness. Matutes scores were really helpful for the diagnosis. In our study, almost all the patients scored 4 or 5, as expected. One patient scored 1, which enabled us to leave CLL aside, since it corresponded to the morphology of a leukaemic phase of lymphoma. The experts' conclusions had no impact on the score which had been predefined by the recruiting centre. Indeed, despite global disagreement in 13% of the cases, the only evolution of Matutes scores was between 4 and 5, both indicating CLL. These differences could be accounted for by the different threshold levels used for the isotypic controls, which modified the percentage of cells considered positive as well as the fluorescence intensity, thus affecting data interpretation. On the whole, global agreement between the three specialists prevailed. The differences of interpretation were not significant. We can thus conclude on the reliability of the laboratories where the immunophenotypes had been performed and say that they are the most capable of interpreting their histograms. Agreement was more frequent with the immunophenotypes than with cytology, which can be explained by the fact that immunophenotypical interpretation is more objective (charts are provided) compared to the relatively subjective dimension of cytologic analysis (“individual” morphologic interpretation).

So as to increase the feasibility of this type of inclusion protocol, we suggest sending the digitized pictures only accompanied with the data related to the Matutes scores as sufficient to validate patient inclusion.

Although no minimal standards of sampling had been pre-established, we found around 40 lymphocytes per file to be appropriate for this type of lymphoproliferative syndrome. This figure is in agreement with the minimum threshold of 30 lymphocytes previously established in another study, which consisted in requesting for specific opinion on various haematological disorders [1]. That figure can be used for both typical and atypical CLL since we have shown that there is no significant difference between the average numbers of captured cells in relation to the complexity of the diagnosis. Moreover, (dis)agreement about the CLL codification between the two experts was not related to the number of captured lymphocytes. Time is another parameter that has to be taken into account in the evaluation of the feasibility of the method. 60 to 90 minutes were necessary to digitize, classify and send the file. We wanted to find out whether there was a connection between the number of pictures taken (and thus the time spent on each smear) and the lymphocytosis. This indeed appears to be true to some extent, and although the tendency was not very clear (“visual” analysis of the slope of the trendline), a statistical link could yet be established. The number of images taken and the lymphocytosis were inversely correlated. Nevertheless, the specialist had to use the maximal magnification available (×100) since analysis of the cellular detail (shape and structure of the cell and its nucleus) is crucial for morphologic subclassification. However, with such a magnification, finding more than one cell in each image proved unfrequent, most images containing only one cell, whatever the cellular density on the smear may have been (high lymphoytoses > 100 × 10^9^/L being included). What is important was to manage to get a sampling which was representative of the smear's morphologic variability (diagnostic criterium). An average of around 40 lymphocytes, whatever the lymphocytosis, seemed to make this possible (practical criterium). A line sampling enabled us to meet these two requirements in most cases.

The time delay between the recruiting centre and the first expert for files sent by post was around one month for one third of the files, which can arbitrarily be defined as “acceptable”. But for the remaining 2/3, the delay was tantamount to several months and even years. One of the causes that could be put forward is that the centres which are requested to participate in cooperative studies are often reluctant to part from their records and archives. The delays and uncertainties caused by sending fragile documents by post indeed represent a serious drawback: the archive materials are frequently deteriorated (broken blood smears) and are not available for quite long periods of time. It was precisely one of the aims of the second reading protocol—namely to avoid parcel sending (at least from an expert to the other) after having certified that the original documents would be returned to the recruiting center once the digitized file was ready. Teletransmission clearly improved the second reading system. However, it is not yet completely satisfying since 16% of the files had still not been validated after a month. That can be explained by a two-week maintenance of the CRIHAN secure website and by the expert's unavailability or material incapability, and so forth. However, electronic data interchange has proved much faster than traditional mail (by post). This is obviously due to a greater speed of transmission (a few seconds instead of several days, or even more). We, however, believe that “motivation” plays a key role and is even more crucial. A direct email enclosing a request for a second reading is probably a greater incentive than more anonymous form letters. The time taken for the expert to reply was, for the most part, compatible with that of clinical decision-making. The expert's role was mainly to give a second opinion (validation). As for traditional second reading (slides sent by post or seminars such as “Forum Workshop”), the slowness of the whole thing prevented the results from being returned on time and, as such, they often came too late to influence in any way the therapeutic options that had already been chosen (for CLLs, a one-month time delay remains acceptable). Thanks to telehaematology, we can thus move on from a hypothetical and dubious retroactive assessment to an upstream quality control of the therapeutic decisions. The notion of time delay could even be eliminated thanks to software that enable two users to establish a direct connection and thus to hold a real-time dialogue and comment upon an image simultaneously thanks to a mobile pointer [5]. For example, that system allowed an expert to guide a nonspecialist technician and thus confirm the diagnosis. But however tempting this solution may appear, it should definitely not be privileged for financial reasons, since this equipment is far more expensive [1, 6]. Indeed, the expert (from where he is) is supposed to perform the tasks that the local morphologist would normally be doing; the expert thus replaces the morphologist instead of merely assisting them. The experts' availability being one of the major parameters accounting for the slowness of telepathology's development, asking them to become substitutes, does not seem very realistic to us [1].

Last but not least, one of the major gains was the creation of an objective database that was available for remote consultation (geographically and temporally). Indeed, one of the aims of the biological protocol was to set up a morphologic database. All the digitized pictures were saved in the CRIHAN secure website and were thus freely available to users, especially in case of dispute over the conclusions. A first step has been taken to solve the semantic inconsistencies related to the use of classifications [7]. The diversity of interpretations now remains to be tackled since, as our study showed, two experts do not necessarily classify the same cell in the same category, even though the differences are almost negligible. The creation of this database also enabled us to keep track of the initial blood smear (which is at present not mandatory in the majority of therapeutic protocols) and thus to keep an eye on disease evolution. As every time information is exchanged, respect of medical secrecy has to be ensured. Using a specifically dedicated talk-back secure website enabled us to guarantee the safety of sent and received messages since passwords were required. All messages posted through the secure website were saved (including the content and the replies) and both document history and traceability were preserved. Consequently, it is essential for good practice to use a dedicated secure website, as we did, rather than traditional emails and the protocol did not call this into question.

We sometimes noticed the difficulties encountered by some recruiting centres to provide us with the files. The time delays sometimes reached months, and even years. Several hypotheses can be put forward: (1) the lack of experience of small-size laboratories in taking part in protocols and the fact that they also keep less track of patient files, (2) the innovative dimension of our protocol, which consisted in assessing the validity of the initial diagnosis, (3) the main (therapeutic) objective of the protocol involved that the specialist was supposed to hand over data to “their” expert so that transmission of the biological files could be properly achieved (the need for which might have been overlooked by the specialist, especially for such a common pathology as CLL). We thus once again would like to stress the importance of establishing a dialogue between the specialists and the experts, from which transmission of the information about the biological and therapeutic protocols would follow. What can also (especially) be called into question is the current chronic underequipment of the laboratories, both in terms of specific hardware and members of staff. The current implementation of teletransmission devices will allow on the premises digitization of the files, which will consequently simplify the procedure and lead to better compliance and efficiency of such types of second reading. Moreover, the conditions regulating resort to “experts” and to “telehaematology expert networks” remain vague in so far as this activity has not yet been officially taken into account by the healthcare authorities and consequently does not benefit from any specific status or any guidelines as far as the financial aspect is involved [8]. There are unfortunately very few documented experiments that we know of dealing with that kind of cytologic activities [4, 9]. We could thus hardly find any data to compare our conclusions with. However, several initiatives implying telehaematology have recently been or are currently being implemented in France (Table 1). Moreover, databases associated with second reading protocols via teletransmission devices have been set up. However, the conclusions of such second readings are not awaited to introduce treatment. City/hospital networks have been created [10]. To finish, there are a few websites, such as Médecin’images or the one of the Collège de Hôpitaux Généraux thanks to which high quality exercises can be performed via teletransmission [11].

## Figures and Tables

**Figure 1:**
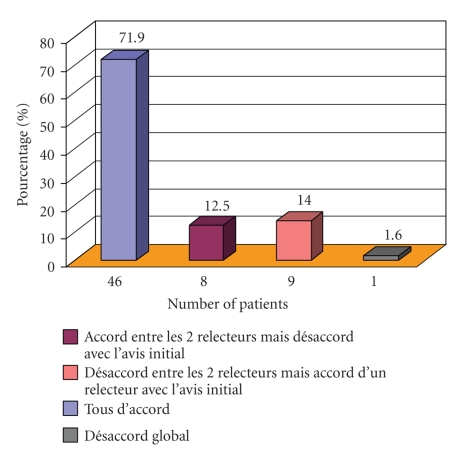
Cytologic concordance.

**Figure 2:**
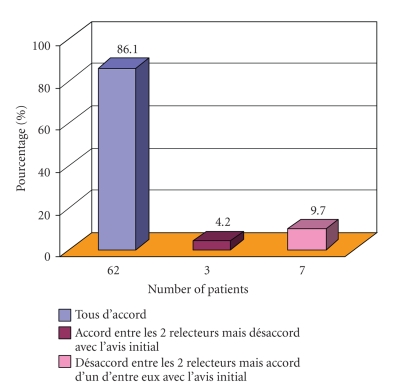
Flow cytometry concordance (Matutes scores).

**Figure 6:**
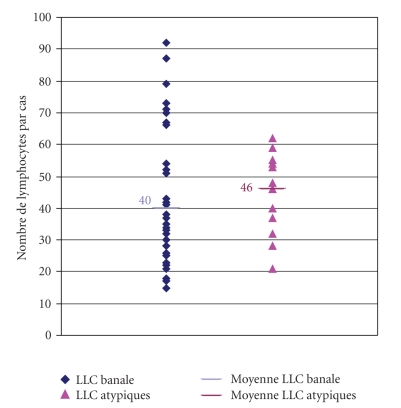
Average numbers of captured lymphocytes functions of the morphologic type of CLL.

**Figure 7:**
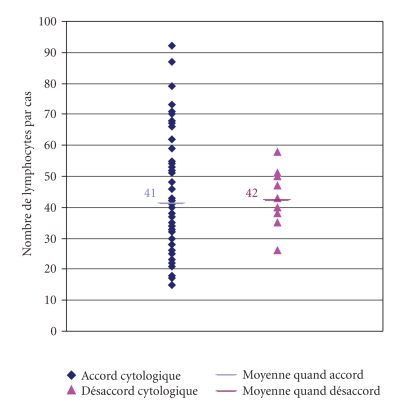
Average numbers of captured lymphocytes functions of cytological concordance.

**Figure 8:**
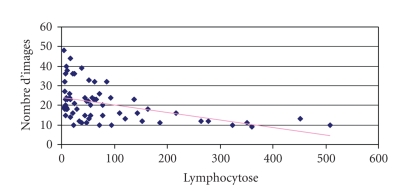
Number of pictures taken functions of the lymphocytosis.

**Figure 9:**
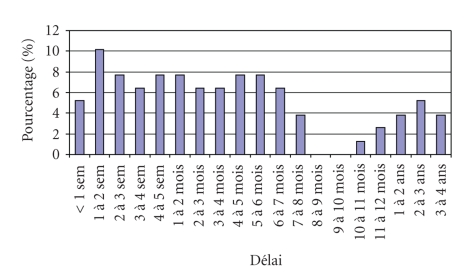
Time delay between initial diagnosis and first review.

**Table 1:** 

Pathology	Protocol	Chair(wo)man	Starting date
*French protocols using telemedecine for mandatory morphological consensus whorkshop (needed for inclusion)*			

Chronic lymphocytic leukemias	GOELAMS LLC 98	JF Lesesve (Nancy) and R Garand (Nantes)	Sept-1999 (achieved)
Acute myeloblastic leukemias (adults)	GOELAMS 3	S Daliphard (Reims) and V Leymarie (Strasbourg)	March-2002 (achieved)
Acute myeloblastic leukemias (adults)	LAM-SA 2002	P Mossuz (Grenoble)	April-2004 (in progress)
Acute myeloblastic leukemias (pediatric)	ELAM02	O Fenneteau (Paris Robert Debré)	March-2005 (in progress)

*French morphological data banks using telemedecine*			

Myelomas	IFM	M Zandecki (Angers)	2000 (achieved)
Acute myeloblastic leukemias	Matchslide	G Flandrin (Paris Necker)	01/11/2001 (achieved)
Red blood cells	Teleslide	G Flandrin (Paris Necker), JF Lesesve (Nancy),	01/09/2003 (achieved)
		O Fenneteau (Paris R Debré), T Cynober (Kremlin Bicêtre)	
Myelodysplastic syndromes	GFMDS	F Picard (Paris Cochin)	Dec-04 (in progress)

*French morphological quality-control tests*			

All topics	Medecin'image	JX Corberand (Toulouse)	
All topics	Collège des Hopitaux Généraux/teleslide	D Lusina, JM Martelli (Aulnay sous bois)	January-2003 (in progress)

*Forum whorkshops (congresses of the “Groupe Français d'Hématologie Cellulaire”) open to discussion*			

Myeloproliferative diseases	Congress GFHC SMP2005, Nantes	R Garand (Nantes)	2005.05.17
B-cell lymphoproliferative syndromes	Congress GFHC SLP2007, Lyon	R Garand (Nantes)	2007.05.22
Acute leukemias and myelodysplastic syndromes	Meeting GFHC, Paris	S Daliphard (Reims)	2006.11.09
Thrombopenia et thombopathies (excluding malignancies)	Meeting GFHC, Paris	S Daliphard (Reims)	2007.11.28
